# Postmastectomy Radiation Therapy: An Overview for the Practicing Surgeon

**DOI:** 10.1155/2013/212979

**Published:** 2013-09-11

**Authors:** Reshma Jagsi

**Affiliations:** ^1^Department of Radiation Oncology, University of Michigan, UHB2C490, SPC 5010, 1500 East Medical Center Drive, Ann Arbor, MI 48109-5010, USA; ^2^Center for Bioethics and Social Science in Medicine, University of Michigan, North Campus Research Complex, 2800 Plymouth Road, Building 16, Room 430W, Ann Arbor, MI 48109-2800, USA

## Abstract

Locoregional control of breast cancer is the shared domain and responsibility of surgeons and radiation oncologists. Because surgeons are often the first providers to discuss locoregional control and recurrence risks with patients and because they serve in a key gatekeeping role as referring providers for radiation therapy, a sophisticated understanding of the evidence regarding radiotherapy in breast cancer management is essential for the practicing surgeon. This paper synthesizes the complex and evolving evidence regarding the role of radiation therapy after mastectomy. Although substantial evidence indicates that radiation therapy can reduce the risk of locoregional failure after mastectomy (with a relative reduction of risk of approximately two-thirds), debate persists regarding the specific subgroups who have sufficient risks of residual microscopic locoregional disease after mastectomy to warrant treatment with radiation. This paper reviews the evidence available to guide appropriate referral and patient decision making, with special attention to areas of controversy, including patients with limited nodal disease, those with large tumors but negative nodes, node-negative patients with high risk features, patients who have received systemic chemotherapy in the neoadjuvant setting, and patients who may wish to integrate radiation therapy with breast reconstruction surgery.

## 1. Introduction

Breast cancer provides an excellent example of how multidisciplinary management has improved patient outcomes. Locoregional control of the disease is the shared domain and responsibility of surgeons and radiation oncologists, and recent evidence demonstrates that efforts to improve locoregional control can indeed influence patients' overall survival [1]. 

Because surgeons are often the first providers to discuss locoregional control and recurrence risks with patients and because they serve in a key gatekeeping role as referring providers for radiation therapy, a sophisticated understanding of the evolving evidence regarding radiotherapy in breast cancer management is critical knowledge for all surgeons who see breast cancer patients. Although some patients receive care from specialized breast surgeons and surgical oncologists who practice alongside consulting radiation oncologists, breast cancer is also often treated by general surgeons who see cases relatively infrequently and who may not practice in settings where informal access to radiation oncologists is so readily available [2]. Therefore, this paper seeks to provide an easily accessible and comprehensive overview of one of the most controversial topics in breast cancer locoregional management: the role of radiation therapy after mastectomy.

Specifically, the paper begins by articulating the theoretical rationale for postmastectomy radiation. It proceeds to detail the early and more recent randomized trials of radiation therapy in this setting. It then turns to criticisms of the various trials and the insights for patient selection that have been offered by retrospective analyses of patterns of failure postmastectomy. It specifically reviews the evidence available to guide patient decision making in areas of controversy, including patients with limited nodal disease, large tumors but negative nodes, and node negative patients with high risk features. The special situation of patients who receive systemic chemotherapy in the neoadjuvant setting, as well as the subject of integrating radiation therapy and breast reconstruction surgery, is also given focused attention. Finally, the paper turns to a discussion of treatment techniques, including considerations for radiation field design, before concluding with reflections on directions for further research.

## 2. Rationale for Postmastectomy Radiation Therapy

It has been known for decades that breast cancer patients can experience locoregional recurrence of their disease in the postmastectomy chest wall or regional nodal basins, including the supraclavicular, axillary, and internal mammary regions. Radiation therapy seeks to eradicate occult disease that remains in these locations, not only to reduce the risk of postmastectomy locoregional recurrence, which is a morbid and distressing event, but also to improve overall survival, by eliminating a reservoir from which distant metastases may be seeded or reseeded. The advent of effective systemic therapies has been postulated to increase the likelihood of eradication of distant micrometastatic disease; if disease in the chest wall or regional nodes is the only disease that remains, its eradication becomes particularly important.

Of course, not all patients have the same risk of harboring residual locoregional disease after mastectomy and systemic therapy, nor of that reservoir being an isolated one. Therefore, a key subject of research has been to identify which patients are likely to benefit from treatment.

## 3. Early Randomized Trials

Early randomized trials of postmastectomy radiation generally demonstrated a reduction in the risk of locoregional recurrence of breast cancer but no clear evidence of impact on distant disease control or overall survival, particularly before the advent of effective systemic therapies [3–6]. As experience with systemic therapy for breast cancer grew, it became increasingly apparent that certain subgroups of patients might harbor a burden of residual locoregional disease that systemic therapies could not eradicate and so might benefit from the administration of postmastectomy radiation therapy. Therefore, a number of trials were initiated to explore the role of postmastectomy radiation in conjunction with chemotherapy [7–9].

For example, in a trial initiated in 1976 by the South-Eastern Cancer Study Group, patients with four of more positive nodes were randomized after mastectomy to six or twelve cycles of chemotherapy (cyclophosphamide, methotrexate, and fluorouracil—CMF) or to radiotherapy followed by six cycles of the same chemotherapy [10]. Although there was a trend towards reduction in locoregional recurrence in the patients who received radiotherapy (relative risk 0.53, *P* = 0.067) but no difference in survival.

Similarly, at the Dana Farber Cancer Institute, patients with positive axillary nodes on a randomized trial of two forms of adjuvant chemotherapy were then randomized after chemotherapy to either postoperative radiotherapy or no further treatment [11]. In an analysis of 510 patients treated on that study from 1974 to 1984, 401 had been eligible for randomization to radiotherapy, of whom 206 had been randomized. Ultimately, 14% of patients randomized to observation after chemotherapy experienced local failure, compared to 5% of those who received radiation (*P* = 0.03), but no differences in distant recurrence or overall survival were observed. The difference observed was mostly driven by failures in patients with four or more nodes involved or level III axillary apical nodal involvement. 

In a randomized trial conducted in Scotland from 1976 to 1982, 322 women aged 70 and under with positive axillary nodes were randomized to postmastectomy radiotherapy alone, chemotherapy alone, or radiotherapy followed by chemotherapy [12]. In that study 12 patients in each radiotherapy arm developed locoregional recurrence, compared to 27 patients who received chemotherapy alone. There was no statistically significant difference in overall or disease related survival between the groups as a whole, but there was a significant difference in disease related survival in patients with 4 or more nodes involved (35% with radiation alone at 5 years, 46% with chemotherapy alone, and 54% with combined modality therapy, *P* = 0.01). 

In the era in which these early studies were designed, the late toxicity of radiation therapy was not fully appreciated, and sophisticated techniques of radiation field design were not yet available. Indeed, many studies initiated in the 1970s and before used older equipment and now obsolete treatment approaches such as large “hockey stick” photon fields that exposed large volumes of the heart and lungs to high doses of radiation. Therefore, it is unsurprising that in meta-analysis of these older studies, the benefits in terms of disease control were ultimately offset by significant treatment-related toxicities. 

A 1987 meta-analysis by Cuzick and colleagues considered 7941 patients entered on trials of radiotherapy after mastectomy [13]. No differences were observed in survival in the first 10 years of followup, but there was an excess of deaths among irradiated patients in trials with followup beyond 10 years, mostly employing radical mastectomy with or without irradiation. A subsequent analysis published in 1994 by the same group went on to find that postmastectomy radiation was associated with improvement in breast cancer cause-specific survival, but that nonbreast cancer events served to offset this benefit [14]. The Early Breast Cancer Trialists Collaborative Group in Oxford, which meets periodically to update its systematic meta-analysis of individual patient data from randomized trials, also demonstrated in its 1995 and 2000 reports, heavily influenced by these earlier trials, a reduction in breast cancer mortality associated with adjuvant radiation therapy that was offset by increases in nonbreast cancer-related mortality [15, 16].

## 4. More Recent Randomized Trials

By the early 1980s, appreciation of the late effects of radiation therapy had progressed, and treatment techniques had evolved. In 1982, the United States Eastern Cooperative Oncology Group initiated a trial of postmastectomy radiation therapy in patients who were at high risk for locoregional recurrence [17]. Specifically, the trial included patients with pathologic T4 lesions excluding T4d disease, T3 lesions with positive nodes, N2 disease (defined as metastases to ipsilateral lymph nodes fixed to one another or to other structures), or earlier stage lesions fixed to underlying muscle. Patients underwent modified or standard radical mastectomy with grossly tumor-free margins and had examination of at least 8 axillary nodes. Patients went on to receive six courses of CAFTH systemic therapy, thus increasing the likelihood that distant micrometastatic disease at presentation might be eradicated, leaving only an isolated reservoir of locoregional residual disease that might be addressed by radiation treatment. Patients on this trial were then randomized to postmastectomy radiation therapy, using relatively modern techniques and doses or observation. Ultimately, in 312 patients who were randomized, after a median followup of 9 years, locoregional recurrence as a component of initial failure was substantially and significantly lower in patients who were assigned to radiotherapy (15% versus 24%). Unfortunately, although the trial was well designed, high rates of noncompliance with treatment assignment and insufficient numbers of patients analyzed with relatively short followup to allow detection of a modest survival difference ultimately limited the impact of the study, which detected no impact of postmastectomy radiation therapy on overall time to relapse or survival [18].

By contrast, trials initiated contemporaneously in Denmark and British Columbia ultimately went on to reveal a substantial benefit from postmastectomy radiation therapy, both in terms of locoregional control and overall survival, and these trials have served as the foundation of existing clinical practice guidelines. These studies included mostly node-positive patients, along with a smaller number of individuals with locally advanced node-negative disease.

Specifically, the Danish Breast Cancer Cooperative Group 82b trial randomized 1789 premenopausal women with high-risk breast cancer (defined as involvement of the axillary nodes, tumor size >5 cm, or invasion of the skin or pectoral fascia), who had undergone total mastectomy and axillary dissection, to CMF chemotherapy and PMRT to the chest wall and regional lymph nodes versus CMF chemotherapy alone [19]. Like the ECOG trial, the inclusion of adjuvant systemic therapy served to increase the likelihood of eradicating distant micrometastatic disease so that improvements in locoregional control could translate into effects on overall survival. Radiation was directed toward the chest wall as well as regional nodes. Specifically, nodal basins irradiated were the supraclavicular, infraclavicular, axillary, and internal mammary nodes in the first four intercostal spaces. Total dose was either 50 Gy in 25 fractions or 48 Gy in 22 fractions. The trial recommended an anterior photon field to treat the supraclavicular, infraclavicular, and axillary regions (with posterior axillary field in larger patients) and an anterior electron field to treat the internal mammary nodes and chest wall. Over 90% of patients were treated using relatively modern megavoltage linear accelerators. 

The landmark results were published in 1997. Analyses of 1709 eligible, randomized patients revealed that postmastectomy radiation therapy yielded not only a substantial reduction in locoregional failure (from 32% to 9%) but also a significant improvement in overall survival (10-year OS improved from 45% to 54%, *P* < 0.001). On multivariate analysis, the study found that primary tumor size, number of involved lymph nodes, grade, age, and use of radiotherapy were all significant independent predictors of outcome; no interactions were observed between radiotherapy and the other characteristics, so that the benefit of radiotherapy was suggested to exist for all subgroups. Moreover, no difference in survival was observed between patients with left and right-sided disease in the initial report at 10 years, and a separate publication considering ischemic heart disease morbidity and mortality revealed no excess risk of ischemic heart disease in irradiated versus unirradiated patients [20]. 

The Danish group also undertook a study in high risk postmenopausal patients. Specifically, in the Danish 82c trial randomized 1460 postmenopausal patients younger than 70 years of age after total mastectomy and axillary dissection, to postmastectomy radiation therapy in addition to tamoxifen for one year or tamoxifen alone [21]. Again, in an analysis of the 1375 eligible patients with a median followup of 119 months, the investigators found that the addition of postmastectomy radiation therapy led to both a reduction in locoregional recurrence (from 35% to 8%) and improvement in overall survival (10-year OS improved from 36% to 45%, *P* = 0.03). Thus, the findings were very consistent with what had been observed in the premenopausal patients on the 82b trial. The examination of interactions between radiotherapy and other prognostic variables this time indicated significantly different effects of radiotherapy depending on number of nodes removed, but only for survival within 4 years. In the first few years after surgery, the beneficial effect of radiation was only observed in patients who had fewer than 8 nodes removed, but with longer followup, the beneficial effects of radiation were the same regardless of the number of nodes removed.

Finally, a smaller trial initiated in British Columbia in 1978 revealed very consistent results [22]. The British Columbia trial randomized 318 premenopausal patients with involved lymph nodes after modified radical mastectomy with axillary lymph node dissection (with a median of 11 nodes removed), to CMF chemotherapy alone or chemotherapy and postmastectomy radiation therapy. Radiation therapy in this trial was administered with Cobalt-60, using 16 fractions to deliver 37.5 Gy with tangential photon fields to the chest wall and 35 Gy to the midaxilla using an anterior supraclavicular and axillary field with a posterior axillary boost. An en face photon field delivered 35 Gy to 3 centimeters of depth to the bilateral internal mammary nodes. After a median followup of 150 months, this trial reported both a reduction in the rates of recurrence and breast cancer mortality from the addition of postmastectomy radiation therapy. Moreover, there were no significant differences in the benefits of radiation therapy on locoregional control or survival free of distant disease between the subgroup of patients with four or more nodes involved and the subgroup with one to three nodes involved. A subsequent update of this study confirmed that there was a significant improvement in overall survival, with 20-year survival improved from 37% to 47% (*P* = 0.03) [23]. Moreover, long-term toxicity was low in both arms, with a rate of cardiac death of 1.8% (3 cases) in the patients treated with chemotherapy and radiation versus 0.6% (1 case) with chemotherapy alone at 20 years (*P* = 0.62). Other toxicities observed included arm edema in 9.1% of radiated patients as compared with 3.2% of unirradiated patients and limited asymptomatic apical lung fibrosis in all radiated patients, but only one patient developing pneumonitis requiring steroid therapy.

Meta-analyses that have included the results of these more recent trials have differed from the earlier meta-analyses discussed previously. For example, one meta-analysis considered 6367 breast cancer patients enrolled in eighteen trials of radiation therapy after mastectomy, axillary dissection, and systemic therapy that were reported between 1967 and 1999 [24]. In this group of mostly node-positive patients, radiation reduced the risk of local recurrence (odds ratio 0.25; 95% confidence interval 0.19–0.34) and overall mortality (odds ratio 0.83; 95% confidence interval 0.74–0.94). Similarly, another meta-analysis revealed an approximately 20% reduction in mortality odds in favor of adjuvant radiation, provided that contemporary techniques were used in order to minimize cardiovascular toxicity [25]. 

Perhaps most influentially, the Early Breast Cancer Trialists Collaborative Group meta-analysis also recently concluded that there was a substantial improvement in local recurrence from postmastectomy radiation therapy in node-positive patients, as well as a modest impact on survival [1]. Specifically, in a landmark 2005 publication, which included data from patients enrolled on trials initiated through 1995, the EBCTCG reported that among 8340 women treated with mastectomy and axillary clearance for node-positive disease and enrolled in trials of PMRT (generally to the chest wall and regional lymph nodes), the five-year local recurrence risk was reduced from 22.8% to 5.8%, with 15-year breast cancer mortality risks of 54.7% versus 60.1% (reduction 5.4%, 2*P* = 0.0002) and overall mortality reduction of 4.4% (64.2% versus 59.8%, 2*P* = 0.0009). This led the EBCTCG to conclude that there was a 4 : 1 ratio, such that for every four local recurrences prevented at five years, one life was saved. 

## 5. Applying the Trial Data: The Controversy over Patients with One to Three Nodes Involved

Despite the strength of these consistent findings from multiple, well-designed randomized trials and their meta-analysis, a number of concerns have been raised about the external validity or generalizability of the Danish and Canadian trials. Concerns include the fact that the systemic therapies administered in the era during which the trials were conducted were not as advanced as treatments available today. The advent of anthracycline-based chemotherapy and aromatase inhibitors, as well as evidence regarding the benefits of longer administration of endocrine therapy, may reduce the level of residual locoregional disease in patients treated with mastectomy in the modern era. In addition, concerns about the adequacy of surgery performed in these trials have in particular led to certain caveats in their interpretation and application to patients treated in the United States in the present day [26]. 

As noted previously, in the Danish 82b and 82c trials, the median number of nodes removed was only 7, and 255 patients on the 82b trial had fewer than 4 nodes removed. This has raised significant concerns that either the surgery performed or the pathologic analysis or both were inadequate. Because so few nodes were examined, there may have been substantial underestimation of the true number of involved nodes, such that patients characterized as having only 1–3 nodes involved in the Danish trials might well have been categorized as having four or more nodes if a more standard and complete level I and II axillary lymph node dissection had been performed. Furthermore, if inadequate numbers of lymph nodes were removed, residual disease in the axilla might have necessitated radiation therapy in a way that might not be the case when a more extensive and complete level I and II axillary lymph node dissection is performed. Indeed, this idea is supported by the finding that the axilla constituted a component of the sites of locoregional recurrence in 13% of unirradiated patients in the Danish trials, [27] contrasting sharply with the much lower rates generally expected from complete level I and II dissection [28, 29]. 

Moreover, numerous subsequently published retrospective series of patients with 1–3 positive lymph nodes after mastectomy and without radiation treatment have demonstrated considerably lower absolute rates of locoregional recurrence than observed in the unirradiated patients on the Danish and British Columbian trials. For example, in 1999, Recht and colleagues reported locoregional failure rates as a function of clinicopathologic features in over two thousand breast cancer patients treated by mastectomy and chemotherapy in four ECOG trials [30]. Locoregional failure was observed in only 13% of cases with 1–3 metastatic nodes, compared with 29% of patients with at least four metastatic nodes. Similar results were reported from a series of over a thousand patients treated with mastectomy at the M.D. Anderson Cancer Center on five trials [31]. In that study, locoregional failure rates were 4%, 10%, 21%, and 22% for patients with zero, one to three, four to nine, and ten or more metastatic nodes, respectively.

In 2005, the British Columbia Cancer Agency published an analysis of the long-term outcomes of 847 mastectomy patients with T1-T2 lesions, and one to three involved axillary nodes who did not receive radiotherapy. The overall risk of developing LRR was 13–16% at ten years [32]. Age less than 45 years, having more than 25% positive nodes, medial tumor location, and ER-negative tumor status were all independently significant factors for LRR and increased the risk from that overall baseline. These findings led the authors to suggest that women with any individual or a combination of these attributes be considered for PMRT, but the risk-benefit ratio for patients without any positive risk factors is low. 

Most recently, in the largest such series to date, Taghian and colleagues analyzed locoregional failures among more than five thousand patients treated on NSABP trials of mastectomy followed by chemotherapy. They observed rates of 13%, 24%, and 32% for patients with one to three, four to nine, and ten or more metastatic nodes, respectively [33]. Given these findings, which diverge substantially from the rates of locoregional failure observed in unirradiated patients with one to three nodes involved in the Danish and British Columbia studies, some have questioned whether the trial results should be applied to this subgroup of patients. 

As a result of these controversies relating to the interpretation and generalizability of the Danish and Canadian trial results, practice in the United States became divided between radiation oncologists who routinely treat and those who routinely observe this subgroup of patients. A 2001 survey of practicing radiation oncologists found that only a modest majority (61.7%) endorsed postmastectomy radiotherapy for patients with one to three involved nodes without extracapsular extension, although 85.2% recommended radiation if extranodal extension was present [34]. A trial was initiated around that time by the North American Intergroup, in which patients with 1–3 nodes were randomized to chest wall and nodal radiation or observation, but the trial unfortunately failed to accrue and closed in 2003.

A number of professional societies convened consensus panels in the United States after the publication of the Danish and Canadian trials, as well as the early retrospective series described previously. These panels generally concluded that the evidence to support PMRT was only strong enough to sustain a recommendation for patients with 4 or more lymph nodes involved; for patients with 1–3 lymph nodes involved, the general consensus of these panels convened a decade ago was that there was insufficient evidence to make suggestions or recommendations for the routine use of PMRT. 

Perhaps the most widely cited consensus guidelines from that era emanated from a multidisciplinary panel of breast cancer experts convened by the American Society of Clinical Oncology (ASCO) Health Services Research Committee for an in-depth review of worldwide data on locoregional failure from breast cancer and the ability of post-mastectomy radiation therapy to reduce risk of locoregional as well as distant relapse [35]. Where evidence-based data was inadequate, the expert panel was charged with utilizing their expert opinion to assess the utility of PMRT. The panel's systematic, graded review of all published evidence regarding PMRT was assembled into a clinical practice guideline. This guideline included recommendations for the routine use of postmastectomy radiation therapy in cases of highest-risk breast cancer, as defined by disease with at least four metastatic lymph nodes. Radiation therapy after mastectomy was suggested for cases of operable Stage III disease. The panel concluded that data regarding net benefits of postmastectomy radiation therapy in cases of T1-2 tumors and 1–3 metastatic nodes were insufficient to make suggestions or recommendations for its routine use. Similarly, the panel assessed the available evidence on using patient factors (such as age or menopausal status) and primary tumor features (such as lymphovascular invasion) and concluded that there was insufficient evidence to make recommendations or suggestions for modifying the guidelines based on these factors. 

Similar attempts at consensus guideline development were undertaken by the American Society for Therapeutic Radiology and Oncology, [36] the American College of Radiology, [37] and the Canadian Committee on Clinical Practice Guidelines for the Care and Treatment of Breast Cancer [38]. Each of these expert panels recommended the use of PMRT in patients with 4 or more positive axillary nodes and acknowledged the controversy surrounding patients with 1–3 positive axillary nodes. However, the ASTRO experts concluded, “The data regarding patient selection for survival advantage are less clear, but the most recent evidence suggests that the greatest survival benefit is seen in node-positive patients with low tumor burdens (i.e., fewer positive nodes or smaller tumors). Radiation therapy in these patients for survival benefit is worthy of consideration, pending more definitive data…. Consultation with a radiation oncologist should occur in node-positive patients treated with mastectomy to help patients assess the risks and benefits of PMRT.” The ACR panel embraced these recommendations. Moreover, since these consensus panels, additional data have emerged to support the role of postmastectomy radiation in patients with 1–3 positive nodes. 

In 2007, investigators from British Columbia and M.D. Anderson Cancer Center published an important collaborative analysis that considered the role of nodal ratios (the ratio of positive to excised nodes) in predicting postmastectomy locoregional recurrence [39]. They found that in patients with 1–3 nodes involved, consideration of nodal ratios helped to reduce differences in estimates of locoregional recurrence between institutions that might relate to the number of nodes removed. They proposed a nodal ratio of >0.20, which predicted a risk of locoregional recurrence exceeding 20%, as a threshold for considering postmastectomy radiation therapy.

Also in 2007, the Danish investigators published a pooled reanalysis of a subset of the patients treated on the 82b and 82c trials [40]. Specifically, they reported on the outcomes of the 1152 node-positive patients with 8 or more lymph nodes removed. They documented a survival benefit of the same absolute magnitude (9%) in patients with 1–3 lymph nodes involved as among patients with 4 or more lymph nodes involved, even though the loco-regional recurrence rates were lower among the former group. This led the authors to debate the EBCTCG's argument for a 4 : 1 ratio between locoregional recurrence prevention and survival, noting that the survival benefit of PMRT is likely related to the ability of systemic therapy to eliminate any existing metastatic deposits at the time of diagnosis. Therefore, they concluded that radiation therapy may be particularly important in the subgroup of patients with less extensive nodal involvement, in whom the burden of distant disease at diagnosis is likely to be less substantial (and potentially more amenable to elimination by systemic therapies) or absent. 

In light of these data taken together, the most recent set of consensus guidelines from the National Comprehensive Cancer Institute states that patients with one to three involved lymph nodes who undergo mastectomy should “strongly consider” radiation therapy.

## 6. Applying the Trial Data: What about Patients with Node-Negative Disease?

Although the Danish and Canadian trials predominantly enrolled patients with node-positive disease, the Danish trials did include patients with large or advanced primaries and node negative. The risk of locoregional recurrence has traditionally been believed to be substantial in patients with node-negative disease who have the relatively uncommon presentation of having large or otherwise locally advanced primary tumors [41, 42]. Therefore, it has seemed reasonable to treat these patients, and the results of the Danish trials supported the idea that not only node-positive patients but also patients with node-negative high risk disease (i.e., tumor size greater than 5 cm and/or invasion to skin or pectoral fascia) benefit from radiotherapy. Among the 135 node-negative patients in the 82b randomized trial, locoregional recurrence was 3% among patients treated with chemotherapy and radiation and 17% in those treated with chemotherapy alone, 10-year disease-free survival was 74% in patients treated with radiation and chemotherapy and 62% in those treated with chemotherapy alone, and 10-year overall survivals were 82% and 70%, respectively [11]. In the 82c randomized trial, among the 132 node-negative patients included, local recurrence as first recurrence occurred in 6% of the patients treated with radiation and tamoxifen and 23% in those treated with tamoxifen alone; 10-year disease-free survival was 43% in patients treated with radiation and tamoxifen and 40% in those treated with tamoxifen alone, and 10-year overall survival was 56% and 55%, respectively [12].

However, more recent evidence has suggested that the risk of locoregional recurrence may be more modest than originally expected in patients with node-negative tumors of 5 centimeters in size or larger. For example, one retrospective study considered 70 patients treated at three institutions by mastectomy and systemic therapy, without radiotherapy, for T2-3,N0 disease (with mean tumor size of 6 cm). In those patients, 5-year locoregional recurrence risk was only 7.6% [43]. Similar findings were observed in an analysis of 313 node-negative patients with tumors greater than or equal to five centimeters (with median tumor size 5.5 cm) who underwent mastectomy but not PMRT in five NSABP trials [44]. Ten-year cumulative incidence of isolated locoregional failure was only 7.1% in this population, and the incidence of locoregional failure with or without distant failure was 10.0%. The investigators were unable to identify any statistically significant prognostic factors for locoregional failure. Given the low incidence of locoregional failure, the authors concluded that postmastectomy radiation might not be indicated for such patients. Of note, few tumors of extremely large size were included in these retrospective studies, so patients with T3N0 tumors still warrant consultation with a radiation oncologist, who may discuss these data with the patient as well as the prospective data demonstrating benefit following the use of PMRT in patients with large or advanced primary tumors and node-negative disease. This may allow for more individualized decision-making.

The decision regarding whether to pursue radiation therapy in patients with borderline T3N0 disease may be further illuminated by some insights gained from retrospective studies of node-negative patients that included patients with smaller primary tumors [45–47]. These studies have identified a number of risk factors for locoregional recurrence in node-negative patients undergoing mastectomy. These risk factors include young patient age, larger tumor size, close or involved surgical margins, presence of lymphovascular invasion, omission of systemic therapy, and high nuclear grade. Specifically, the International Breast Cancer Study Group conducted a retrospective analysis of 1275 node-negative patients treated in their IBCSG trials that suggested that while overall locoregional recurrence rates were low, risk was substantial in certain subgroups with multiple risk factors. These risk factors included vascular invasion and tumor size greater than 2 cm in premenopausal patients and vascular invasion in postmenopausal patients. Similarly, in a retrospective analysis of 877 node-negative postmastectomy patients treated at the Massachusetts General Hospital the overall cumulative incidence of local-regional recurrence at ten years was only 6.0%. However, a number of risk factors appeared to predict for locoregional failure, including (1) tumor size greater than 2 centimeters, (2) premenopausal status, (3) margins less than two millimeters, and (4) evidence of lymphovascular invasion. In addition, in a series of 1505 women with T1-2N0 breast cancer treated with mastectomy alone in British Columbia, the ten-year locoregional recurrence rate was 7.8% overall, but higher rates were observed in subgroups with multiple risk factors, including grade, lymphovascular invasion, tumor stage, and absence of systemic therapy. Therefore, it may indeed be useful to consider these factors when deciding whether a patient is likely to benefit from postmastectomy radiation therapy.

Particular attention has been given to the implications of close or positive surgical margins. A number of retrospective studies have addressed this issue. For example, investigators at Fox Chase Cancer Center analyzed patterns of failure in 34 patients with close or positive surgical margins after mastectomy, who did not receive radiation therapy, from a cohort of 789 patients treated by mastectomy between 1985 and 1994 [48]. Two patients had positive margins, and 17 had margins of 2 mm or less. Only 5 chest wall failures occurred, exclusively in patients aged 50 or younger, and this subgroup had a risk of 28%, leading the authors to advocate radiation therapy for margin status in this subgroup. Another more recent study from British Columbia analyzed patterns of failure in the 94 patients with positive margins observed in a cohort of 2570 women with T1-2N0 invasive breast cancer treated with mastectomy. Of these 94 women, 41 had received radiation and 53 had not. Trends for higher risk of locoregional recurrence were observed in the absence of radiation therapy for patients with age under 50 years (20% versus 0%), T2 tumor size (19.2% versus 6.9%), high grade disease (23.1% versus 6.7%), or lymphovascular invasion (16.7% versus 9.1%). The authors observed no locoregional relapses in patients with age over 50 with T1 tumors that were Grade I or II without lymphovascular invasion, leading them to conclude that these data support the “judicious, but not routine use of PMRT for positive margins after mastectomy in patients with node-negative breast cancer [49].”

Of note, even patients with pure DCIS may have close or involved surgical margins, and whether this feature indicates a substantial enough risk of locoregional recurrence to merit radiation therapy has been a subject of considerable recent attention. In a series from Southern California of 574 patients who underwent mastectomy for pure DCIS, 84 patients with margins closer than 10 mm were evaluated [50]. Of these, 31 had margins of 2 mm or less, and 5 of these (16%) experienced local recurrence, compared with only 1 (2%) of the 49 patients with margins between 2 and 10 mm. All recurrences occurred in patients under 60 years of age. These authors concluded that postmastectomy radiation might be indicated for this group. However, several subsequent studies have suggested that risk of chest wall recurrence after mastectomy for DCIS is low even in patients with close or positive margins. One of these analyses considered 55 patients with close margins and 4 patients with positive margins after receiving mastectomy for DCIS at the University of California at San Francisco, finding that the risk of chest wall recurrence was only 1.7%, and 3.3% among high-grade patients alone [51]. This led the authors to recommend against postmastectomy radiation for close margins after mastectomy for pure DCIS, although they had too few patients with positive margins to draw any conclusions about that group. An analysis of 142 patients treated at Faulkner Hospital in Massachusetts revealed chest wall recurrence rates of 1.4% overall, 4.8% (1/21) for those with positive margins, 4.3% (1/23) for close margins of 2 mm or less; and 0 (0/98) for those with negative margins [52]. Again, these authors concluded that the rates of recurrence were so low that radiation therapy was not warranted.

## 7. Advances in Understanding the Role of Tumor Biology

In recent years, the field of oncology has begun to appreciate that breast cancer is a heterogenous disease, in which tumor biology can be at least as important as clinicopathologic stage in determining outcomes. Therefore, interest has been growing in the evaluation of outcomes by biologic subtype, as well as in defining genomic predictors of recurrence.

The Danish investigators have published an interesting analysis of their trial results by biologic subtype [53]. Tissue microarray sections from 1000 patients treated on the DBCG 82b and 82c trials were stained for ER, PR, and Her-2. Significantly improved survival was observed from PMRT in patients within the best prognostic subgroups—those with positive hormone receptors and negative for Her2; in contrast, patients in the poorest prognostic subgroup of patients, whose tumors were negative for hormone receptors and overexpressed Her2, no survival benefit was observed. However, it is important to note that the Danish trials were conducted in an era prior to the advent of trastuzumab therapy for patients with Her2 positive disease, and so these results are difficult to apply to patients treated in the modern era. The authors conclude that there may have been distant unresponsive micrometastases in patients with more aggressive biologic subtypes for which systemic therapy at the time of the trials was inadequate. 

Several groups have evaluated locoregional recurrence risks after mastectomy in triple negative patients, whose tumors are negative for hormone receptors and Her2. In a large population-based cohort from Alberta, investigators found a substantial enough rate of locoregional recurrence in node-negative, triple-negative breast cancer patients undergoing mastectomy that they suggested consideration of postmastectomy radiation in that subgroup [54]. At the same time, a trial from China was published that suggested a substantial benefit from postmastectomy radiation in triple-negative, node-negative patients [55]. However, given the unexpectedly large magnitude of the benefit observed, questions remain regarding the quality control of diagnostic and therapeutic modalities in that study, and most practitioners do not routinely recommend postmastectomy radiation in this subgroup at this time. Moreover, the Danish study discussed previously suggested that triple negative patients may be less radiosensitive, and several institutional reviews of patients receiving radiation after breast conserving surgery have also raised this concern. Clearly, further research is necessary to help tailor locoregional therapy by tumor biology.

Investigators from Duke and Taiwan have made interesting advances in developing a platform for genomic profiling to predict locoregional recurrence risk after mastectomy and the need for postmastectomy radiation [56]. In a tissue microarray study of 94 breast cancer patients who underwent mastectomy without radiotherapy, these authors identified two gene expression profiles that were predictive of locoregional recurrence. Ongoing efforts along these lines may ultimately allow for the more tailored selection of patients most likely to benefit from treatment.

## 8. Patients Receiving Neoadjuvant Chemotherapy

An increasing number of patients treated today receive preoperative systemic chemotherapy. Studies have shown that survival is not compromised by this approach, and administration of systemic therapy preoperatively can improve the chances of ultimately being able to achieve breast preservation. Moreover, patients who are conflicted about their surgical decision may welcome the additional time that neoadjuvant therapy affords, especially if genetic testing results are awaited. Unfortunately, the trials of postmastectomy radiation therapy were conducted in patients who received the more traditional sequence of surgery followed by systemic therapy. Therefore, much of our understanding of the role of radiation therapy after mastectomy relies upon pathologic staging that was conducted prior to exposure to systemic therapies. Extrapolating from those data to the situation of patients who have received systemic therapy prior to definitive pathologic evaluation has been complicated.

Retrospective studies from the MD Anderson Cancer Center have provided a number of insights regarding the role of radiation therapy in patients undergoing mastectomy after neoadjuvant chemotherapy. For example, a 2004 study from that institution examined the ten-year outcomes of 542 patients treated with neoadjuvant chemotherapy, mastectomy, and radiation therapy [57]. Investigators compared these patients to a control group of 134 patients who did not receive radiation, finding that irradiated patients had significantly lower rates of locoregional recurrence (11% versus 22%). Moreover, even patients who experienced a pathologic complete response to neoadjuvant chemotherapy had a substantial reduction in locoregional recurrence risk, if they had initially presented with clinical Stage III. At ten years, these patients had a 3% LRR rate (1 event among 35 patients) versus 33% (3 events among 11 patients) in nonirradiated patients. Post-mastectomy radiation also appeared to improve cause-specific survival in patients with Stage IIIB disease, clinical T4 tumors, and greater than four positive lymph nodes. Although retrospective studies are subject to standard caveats regarding selection bias, one might have expected the patients who received radiation to be among the highest risk subgroup. Thus, these findings were highly suggestive of benefit in patients presenting with Stage III disease.

A subsequent analysis of the same patients from M.D. Anderson was published in 2005 to examine the risk factors associated with LRR. This study revealed the importance of disease staging both before and after neoadjuvant chemotherapy, as several risk factors were associated with either the pretreatment or posttreatment extent of disease. Supraclavicular nodal involvement on presentation was associated with a higher risk of LRR after treatment. On postneoadjuvant chemotherapy assessment, evidence of skin or nipple involvement and extracapsular invasion were also strongly correlated with LRR. Lack of tamoxifen use postoperatively was also associated with increased LRR, but due to the preponderance of ER-negative patients with increased LRR, it was felt to be of little clinical significance. Of note, ER-receptor negative disease was the strongest predictor of LRR in this group. Patients with one or none of these factors had a 4% ten-year LRR rate, but this rate jumped to 28% with the presence of three or more risk factors [58]. 

In contrast to the substantial risk of locoregional recurrence observed in patients with more advanced clinical presentations who did not receive postmastectomy radiation, retrospective studies have also highlighted a group in whom the risk of recurrence appears relatively low: patients presenting with clinical Stage II disease and whose disease responds to neoadjuvant therapy. Specifically, in a series from M.D. Anderson, no locoregional recurrences were observed in a small number of patients who achieved pathologic complete response after diagnosis with clinically early-stage disease [59]. 

More recently, Mamounas and colleagues from the NSABP have provided additional information from retrospective analysis of locoregional recurrence patterns in patients treated on the report the NSABP B18 and B27 randomized trials of preoperative systemic therapy [60]. Patients on these trials had operable cT1–T3, N0-1 breast tumors and received chemotherapy (doxorubicin/cyclophosphamide alone or followed by neoadjuvant/adjuvant docetaxel) prior to definitive surgery to the breast and axillary lymph node dissection. The majority of patients then received lumpectomy (64%), but 36% received mastectomy (36%). Of particular importance is the fact that patients who underwent mastectomy received no radiation per protocol on these trials. This consistent approach makes these data highly valuable for documenting rates of locoregional recurrence in the absence of radiation, since patients were not selectively radiated. Ten year locoregional recurrence for the group of patients receiving mastectomy was 12.6%. However, in the small number of patients with pathologic complete response, only 1 recurrence was seen in 94 patients, regardless of tumor size and clinical nodal status. Thus, the findings of these retrospective analyses have raised questions regarding the need for postmastectomy radiation in the subset of operable node-positive patients who experience a robust response to neoadjuvant systemic therapy.

In light of these issues, the National Cancer Institute convened a multidisciplinary expert panel in 2008 specifically to advise on locoregional management of patients receiving neoadjuvant systemic therapy [61]. The group published a scientific consensus statement that concluded that PMRT should be considered for patients presenting with clinical Stage III disease or with histologically positive lymph nodes after preoperative chemotherapy. It further concluded that there was a need for prospective trials to evaluate the benefits of PMRT in patients with clinical Stage II disease who have negative lymph nodes after preoperative chemotherapy. 

Given the complexities of assessing risks based upon prechemotherapy clinical data and postchemotherapy pathology in patients treated with neoadjuvant chemotherapy, treatment in a multidisciplinary context is especially important in these cases. Early involvement of a radiation oncologist in multidisciplinary consultation, preferably prior to the commencement of chemotherapy, may be particularly useful. 

## 9. Defining the Appropriate Targets of Radiotherapy

No discussion of postmastectomy radiation therapy would be complete without a discussion of the appropriate targets of treatment. As noted previously, the landmark trials from Denmark and British Columbia included radiation treatment to not only the chest wall but also the supraclavicular, axillary, and internal mammary nodal regions. However, some of the concerns discussed previously regarding the extent of nodal surgery performed in these trials, as well as the information on patterns and locations of locoregional failures from retrospective studies, have led to some debate over the optimal targets of radiation treatment in patients who have undergone mastectomy. There is little debate that the chest wall, which is the highest risk site of failure, [62–64] is a key target of postmastectomy radiation. However, attitudes are more divided regarding which regional nodal basins merit coverage.

In the United States, where axillary lymph node dissections are considerably more extensive than in those trials, and where the risk of axillary recurrences is extremely low, there has been general consensus that directed radiotherapy to the axilla is generally unnecessary, although it may be considered in cases where the nodal dissection is less extensive, or in select cases of extensive nodal involvement or gross extranodal extension that raises the concern of residual microscopic disease in that region. 

There is less consensus regarding the situations in which one should treat the supraclavicular and internal mammary nodal regions. Individual institutions have published excellent outcomes in patients treated to the chest wall alone for T1-T2, N1 disease. However, recent preliminary data from the National Cancer Institute of Canada's MA20 trial have provided indirect support for the inclusion of treatment to these regions. Specifically, in MA20, 1832 patients who underwent breast conserving surgery were randomly assigned to whole breast radiation therapy alone or whole breast radiation in conjunction with treatment to the regional nodes. Preliminary results suggest a 5.4% absolute improvement in distant disease-free survival and a 2.3% benefit in locoregional disease-free survival from the addition of radiation therapy to the supraclavicular and internal mammary regions. Because there is no reason to believe that the regional lymph nodes have a different likelihood of harboring an isolated reservoir of locoregional disease in patients who undergo mastectomy rather than lumpectomy, the results of MA20 have been taken by many to support treatment to these fields in the postmastectomy setting as well.

Of course, the MA20 trial did not isolate the impact of supraclavicular versus internal mammary radiation, nor did the EORTC 10925 trial, which still remains to be analyzed. Supraclavicular fields are generally less controversial, given that a nontrivial minority of failures occur in this region, [65] and treatment is believed to result in little if any increase in the risks of pneumonitis, brachial plexopathy, and lymphedema. In contrast, concerns about the potential cardiac and pulmonary toxicity associated with treating the parasternal internal mammary nodal region are significant. This has led to particular controversy surrounding treatment to the internal mammary region in particular, [66–68] and widespread variation in practice patterns [69]. Considerable controversy remains regarding the need to treat this region, and practice varies widely. 

However, the internal mammary lymph nodes are an important potential site of breast cancer locoregional extension. Early studies of extended radical mastectomy documented involvement of the internal mammary nodes in many breast cancer patients, particularly in those with involved axillary nodes and central or medial tumor location [70–75]. Of course, those studies included patients with more advanced disease than typically diagnosed in the modern era. Still, even relatively recent lymphoscintigraphy studies have revealed drainage to the IMNs in many cases. Internal mammary drainage is more common with central or medial tumors, and patients with parasternal drainage and involved axillary nodes have been found to have substantial rates of pathologically confirmed internal mammary nodes as well. Although internal mammary nodal recurrences are rare, this may well be an artifact of the fact that patients do not undergo routine surveillance of this region in followup.

Recently, a French randomized trial was reported that was initiated to assess the impact of postmastectomy radiation therapy, with or without treatment specifically targeting the internal mammary region, in patients with positive axillary nodes or with negative nodes but a central or medial primary tumor [76]. Unfortunately, the trial was designed prior to the availability of the results of the Danish and Canadian trials mentioned previously, and it was powered to detect a 10% difference in overall survival from internal mammary treatment, an unrealistic goal, given the magnitude of the overall survival benefit of postmastectomy radiation therapy as a whole. Moreover, its inclusion of node-negative patients, in whom the risk of IMN involvement is lower, further decreased the ability of the trial to detect a meaningful difference in a more appropriately selected, higher risk population. Given the use of outdated, two-dimensional treatment planning techniques and a lack of quality control, the trial ultimately yields little insight into the role of internal mammary radiation as a component of modern postmastectomy radiation therapy. In contrast, a recent retrospective observational study from Korea has suggested that there may indeed be benefit from radiation to the internal mammary region specifically when treatment is administered with three-dimensional planning [77]. 

In any case, at this time, guidelines conclude that there is insufficient evidence to recommend for or against internal mammary nodal treatment and leave the decision to the treating physician. I have previously advocated for individualized consideration of the risk of IMN nodal involvement, as well as patient anatomy and ability to exclude critical normal structures from the treatment fields [78]. While it does not seem prudent to treat the internal mammary region when the risk of involvement is low, such as cases of micrometastatic axillary disease, coverage of this region does seem worthwhile when axillary involvement is more substantial, particularly when the tumor is medially located or other high-risk features exist. With modern techniques, including consideration of respiratory gating, it appears possible to cover the IMNs in most patients with very low dose to the heart and its critical coronary vasculature. Given the totality of the existing evidence, it seems reasonable to encompass the internal mammary region in postmastectomy treatment in patients who have substantial risk of involvement and in whom coverage can be achieved with only low-dose scatter to the heart. 

## 10. Radiation Treatment Techniques

As discussed previously, the advent of new technologies that have facilitated the more conformal treatment of the targets of postmastectomy radiation therapy have been essential in allowing the improvement in breast cancer disease control to translate into an impact on overall survival. Detailed description of radiation treatment techniques is outside the scope of this paper, which is intended to provide an overview of the relevant issues for the surgical practitioner. However, this section provides a brief description of the usual approach in the modern era.

As described in detail elsewhere, modern treatment techniques generally utilize three-dimensional planning and cover the chest wall using tangent beams of high-energy photons generated by a megavoltage linear accelerator [79]. The supraclavicular and infraclavicular regions may be treated with an anterior photon field (usually obliqued slightly medially to avoid the spinal cord), and care is taken to avoid overlap with the tangent fields, using beam blocking or couch angles to address beam divergence. If the axilla requires treatment, a posterior axillary boost field may be used to provide adequate dose at depth, given that the axillary nodes are situated deeper than the supraclavicular and infraclavicular nodes. 

If the internal mammary nodes are targeted, a number of techniques are available [80, 81]. One favored approach is a “wide” or “deep” tangent technique, in which the medial border of the tangent field is placed beyond the midline to allow the parasternal region at the levels of the first three intercostal spaces to be included within the tangential fields, with blocking inferiorly to exclude the heart and lung. If this technique does not allow for sufficient blocking of normal structures, as determined by analysis of a dose-volume histogram or normal tissue complication probability model, a separate electron (or mixed photon/electron) beam may be employed. More advanced techniques of intensity modulation [82] and proton beam treatment [83] are also under investigation. After treatment to the chest wall and regional nodes, usually to a dose of approximately 50 Gray in 2 Gray per fraction daily, five days a week, a boost may be administered to the mastectomy scar. This is based on extrapolation from clinical trial data in the setting of treatment to the conserved breast, as well as on retrospective data suggesting potential benefit. The chest wall scar boost is generally accomplished using en face electrons. 

## 11. Complications of Treatment

As with any therapy, one must carefully weigh the potential benefits against the risks for toxicity. Radiation treatment can result in a number of acute and late toxicities. Acute toxicity in the context of postmastectomy radiation therapy is generally limited to fatigue and a skin reaction. Because the skin is a target of treatment in the postmastectomy setting, erythema is expected, and patients can develop a more severe reaction of dry or even moist desquamation that can be painful and disturbing, requiring management similar to that of a thermal burn. 

A subacute reaction that develops in a small proportion of radiated patients is radiation pneumonitis [84]. An inflammatory process that develops several weeks after the completion of radiation therapy, pneumonitis appears to occur more frequently in patients who have a larger volume of lung irradiated, as well as receipt of certain systemic agents [85, 86]. Careful treatment planning to minimize the proportion of lung irradiated helps to keep the risk of pneumonitis in recent studies quite low [87]. Fortunately, radiation pneumonitis tends to respond well to steroid therapy and rarely results in lasting, clinically significant pulmonary damage.

Chronic complications can be the consequence of fibrosis or late changes in any of the normal tissues incidentally irradiated. Although lymphedema may occur after axillary dissection alone, the risk appears to be increased by the addition of radiation therapy to the regional nodes [88–90]. Brachial plexopathy has been reported, although it is exceedingly uncommon at the doses and standard fractions usually used to treat breast cancer in the United States in the modern era. Costochondritis or rib fractures may occur in some patients following the completion of radiation therapy.

Radiation is known to induce secondary malignancy, although quite uncommonly. The excess cancer incidence mainly appears to involve a low increase in the risk of contralateral breast cancer and lung cancer among patients receiving radiation therapy for breast cancer, as detailed in the EBCTCG meta-analyses. Radiation-induced sarcomas have been described, although they are extremely uncommon, with a cumulative incidence of 2 to 3 cases per thousand at 10 years [91, 92].

The potential cardiotoxicity of radiation therapy has been and continues to be the source of considerable concern. As discussed in detail previously, the cardiotoxic effects of older treatment techniques, which exposed large volumes of the heart to high doses of radiation, have been clearly established [93]. More recent studies have raised concerns about even more conformal, modern techniques. In particular, concerns relate to the fact that the left anterior descending coronary artery may be incidentally irradiated to high doses by chest wall tangents. The right coronary vessels can also receive substantial dose when the internal mammary region is targeted. Reassuringly, population-based studies have suggested that the magnitude of increased cardiac risk related to radiation therapy has decreased in more recent years [94]. However, it is sobering that perfusion defects (for which the clinical consequences have yet to be defined) have been observed even in patients treated with relatively modern techniques [95]. Several single-institution studies have suggested that there may be an increase in the relative risk of ischemic cardiac events following radiation therapy for left-sided breast cancer, although the absolute magnitude of this increased risk appears to be low [96]. Recent studies have also suggested radiation and other cardiac risk factors, such as hypertension or smoking, may be synergistic in their effects [97, 98]. A recent population-based case-control study has highlighted the importance of minimizing dose to the heart [99]. However, it is important to note that the net survival benefit of radiation therapy that was documented in the trials discussed previously and in the meta-analysis already accounted for any adverse impact of cardiac toxicity on survival. Therefore, although further reduction in dose to cardiac structures is a worthy and important endeavor, patients with substantial likelihood of net benefit should not avoid treatment solely due to concerns related to cardiac exposure.

## 12. Integrating Radiation Therapy and Breast Reconstruction

Breast reconstruction has been increasingly recognized to play an important role in the quality of life of many breast cancer patients who undergo mastectomy. Since the Women's Health and Cancer Rights Act mandated insurance coverage of breast reconstruction in the United States, an increasing proportion of women have undergone reconstruction [100]. Because of the accumulating evidence described previously regarding the benefits of postmastectomy radiotherapy in select patients, this means that a growing number of patients must make complex decisions regarding the integration of these two treatments. Reconstruction can involve a variety of techniques, utilizing the patient's own tissues or synthetic implants, sometimes with a temporary tissue expander placed first and later replaced. Reconstruction can be completed at the time of mastectomy, it can be initiated with tissue expander placement, or it can be delayed entirely until the completion of radiation therapy. Patients and physicians making decisions regarding type and timing of reconstruction must therefore consider the potential impact of radiation on outcomes with these different approaches.

Unfortunately, when the chest wall or reconstructed breast is irradiated, changes may result in the treated soft tissues and skin that may compromise the viability and cosmetic outcome of reconstruction, potentially causing morbidity and distress and requiring repeated procedures for correction. These risks may relate to the type and timing of reconstruction, but high-quality evidence regarding these issues is relatively limited.

Patients receiving implant-based reconstruction can develop complications that include scarring at the interface between implant and tissue, capsular contracture, infection, pain, skin necrosis, fibrosis, and impaired wound healing. These risks do appear to be higher in patients who receive radiation [101–104]. Patients who undergo reconstruction with autologous tissue techniques are at risk for fat necrosis, fibrosis, atrophy, and flap contracture, [105, 106] but certain studies have suggested that this technique yields better results in conjunction with radiation than implant-based techniques [107]. 

The timing of treatments may be particularly important in this context. After all, while the majority of patients who receive breast reconstruction do so at the time of the initial mastectomy (so-called “immediate reconstruction”), a minority do so as a separate surgical procedure (“delayed reconstruction”). Some believe that immediate reconstruction is particularly suboptimal in the setting of radiotherapy because it leads to the exposure of the full reconstruction to radiation, and some studies have suggested particularly increased complication rates in this setting, [108] but certain single institutions have documented excellent outcomes with carefully standardized regimens [109]. Immediate reconstruction does complicate radiation treatment planning, and it is even possible that recurrence rates could be affected [110, 111].

Ultimately, the challenge in this area is that no randomized trials have evaluated the impact of radiation therapy, or differences in techniques or timing of treatment, on complications or cosmetic outcomes of breast reconstruction. In fact, most evidence regarding the effects of radiation therapy in this setting is limited to the findings of observational series, most of which are small, retrospective, and limited in other ways. These limitations include use of nonstandardized outcomes measures and frequent absence of information on critical factors known to correlate with surgical complications, such as diabetes, body-mass index, or type of implant. Unsurprisingly, given these limitations, estimates of the rates with which complications develop in patients who do and do not receive radiotherapy vary substantially between different institutional series.

Several high-quality studies have been published in recent years from a number of prominent institutions, supporting excellent outcomes with their particular approaches, which vary quite a bit from one another [109, 112]. However, the ability to extrapolate from these experiences to the broader community is challenging. A few multicenter studies have been conducted as well. The Michigan Breast Reconstruction Outcomes study unfortunately lacked adequate numbers of radiated patients for evaluation [113, 114]. It did demonstrate, however, that patients undergoing reconstruction have substantial rates of major complications even in the absence of radiotherapy—31.6% of patients experienced hospitalization, reoperation, or needed intravenous antibiotics within the first two years of reconstruction. A French multicenter study recently evaluated the factors associated with reconstruction failure and capsular contracture in 141 patients who received mastectomy and implant reconstruction, followed by radiotherapy [115]. It found grade 3 or 4 capsular contracture in 32.5% of cases, with 32 cases of complications that required surgery. Unfortunately, lack of an unirradiated control group and absence of analysis of factors known to correlate with surgical complications limited the impact of the study [116]. 

In sum, given the limitations of the existing evidence, decisions regarding the best approach to sequencing and techniques in patients receiving both radiation and reconstruction continue to be based largely on local or institutional traditions and anecdotal experience. This remains an area of active debate and ongoing research.

## 13. Conclusions and Directions for Future Research

As reviewed previously, considerable evidence has been collected regarding the role of radiotherapy after mastectomy, and the subject has been one that has generated extensive controversy over the past few decades. Although substantial evidence indicates that radiation therapy can reduce the risk of locoregional failure after mastectomy (with a relative reduction of risk of approximately two-thirds), debate persists regarding the specific subgroups who have sufficient risks of residual microscopic locoregional disease after mastectomy to warrant treatment with radiation. The optimal targets of treatment and techniques to minimize toxicity, particularly in patients who also wish to pursue reconstruction, continue to be the subjects of ongoing debate.

Trials are ongoing to evaluate many of these issues. For example, studies continue to seek to refine the ability to select patients for radiation treatment and evaluate the role of postmastectomy radiation therapy in patients with limited nodal disease or high-risk node-negative disease. The SUPREMO study, led by the Medical Research Council in the United Kingdom, closed to accrual in 2013 and included patients with T1-2, N1 disease, or T2N0 disease with high risk features such as high grade or lymphovascular invasion [117]. Trials will also soon begin to explore the role of postmastectomy radiation therapy in patients who have pathologically involved axillary lymph nodes prior to neoadjuvant chemotherapy but whose nodal disease is sterilized by the time of surgery. The proposed NSABP B51/RTOG 1304 study focuses on this issue. 

Ongoing research also seeks to explore the role of advanced technologies in reducing cardiac and pulmonary exposure, as well as to identify the appropriate regional nodal basins for coverage and the best approaches for combining radiation therapy and breast reconstruction. Final results are eagerly anticipated from the Canadian NCIC MA20 study, as well as the European EORTC 22922/10925 study, which both focus on the role of supraclavicular and internal mammary regional radiation. An ongoing Korean KROG study focuses on internal mammary radiation specifically. Studies also seek to identify more sophisticated genomic predictors of locoregional recurrence in order to improve patient selection for therapy.

Ultimately, these efforts will allow us to further individualize therapeutic recommendations for breast cancer patients. But in the meantime, the existing data strongly support radiation therapy at least in patients with advanced nodal disease. Recent studies suggesting that radiation therapy is still substantially underutilized in mastectomy patients meeting these criteria are worrisome [118, 119]. Heartening are data that suggest that surgeon participation in breast cancer patients' decisions regarding postmastectomy radiation therapy is strongly associated with appropriate receipt of treatment. Therefore, it is critically important for all surgeons who treat breast cancer to consider the evidence reviewed in this paper, in order to ensure that their patients receive appropriate guidance.
